# Diseases of the Blood

**Published:** 1904-10-29

**Authors:** 


### DISEASES OF THE BLOOD.

(*Concluded from page* 70.)

**Uncinariasis.**—L. M. Warfield[24] shows the importance of the discovery that much of the anæmia prevalent in the Southern States of America is due to "hook-worm" disease and "ground-itch." This has hitherto been ascribed to malaria and "miasma," and has largely reduced the productiveness of the population. He demonstrated the ova of the worm in the stools, and found that in almost all cases of hook-worm there had been previous attacks of ground-itch. The connection is confirmed by Bentley's analogous discovery of ankylostoma in the water-itch of Assam. The blood state is nearly like that of pernicious anæmia, but shows an excess of eosinophiles. At times a long-continued fever may be present, or acute attacks of febrile character. The diagnosis depends on the discovery of the eggs in the stools, which is extremely easy, since a smear shows several in the field under a low power without the trouble of staining or fixing. For treatment a dose of salts may be given in the evening, and no food afterwards till noon next day; at 8 A.M. 30 grains of thymol are given in water, or a capsule; at 10 A.M. a second dose of the same amount, followed by a large dose (℥i. to ℥ii.) of castor-oil or salts. Other writers warn us against the risk of giving castor-oil or alcohol, lest they combine with the thymol and produce poisonous effects.

**Ankylostoma.**—In Europe we are threatened with a similar anæmia due to the ankylostoma duodenale. Southern Europe has already been widely infected, and outbreaks have occurred in Cornwall and Scotland. In Westphalia 17,000 men were found infected in certain collieries,[25] and though there is some protection in England from the coldness of the climate, which prevents the hatching of the eggs out of doors except in summer, outbreaks have occurred as far north as the brickfields of Cologne. In warm damp mines in this country the disease spreads rapidly, and causes profound anæmia with disturbances of the stomach and bowels, and sometimes fatal results. Haldane shows that infection is carried not only through larva in contaminated food and water, but probably also by an infection of the skin, accompanied by an eruption known in Cornwall as "bunches." The worms themselves are whitish, about half an inch long, and have two pairs of hooks at the mouth. The eggs as in the American variety are easily found in the fæces, and the treatment is by similar doses of thymol until the stools are free from eggs. Prevention of any infection in mines is of the utmost importance, and to this end various regulations and careful inspection are necessary.

**The Blood in General Paralysis.**—Diefendorf,[26] neglecting the work of those who had not the advantage of modern instruments, found that the results of a careful and extensive study of the blood, from the earliest stages to the termination, in 11 cases of this disease, agreed in general with those of other recent observers; viz., Macphail (1889); Thompson and Bevan Lewis, and more recently still, Smyth (40 cases); Winkler (4); Kryspiakiewier (15); Roncoroni (15) (who studied the eosinophil cells and found them rarely normal, sometimes diminished, sometimes increased, and in one case amounting to 25 per cent.), Burton (4), Somers (5), Capps (19), and Jeliffe (17). The author's observations show that there is in the first stage anæmia, never very great, but increasing as the disease advances; that there is an increase of hæmoglobin at the onset of the terminal stage, but that during the intermediate stage there is no increase, though the patient's condition improves with the care usually afforded at this time by an institution. The number of red cells is normal, or a little less than normal, decreasing slightly until the final stage, when the number rises again concurrently with the increase in hæmoglobin. Thus a loss of weight and impairment of nutrition is accompanied by an increased richness of the blood. No changes are noted during the paretic states of excitement, stupor, or quiescence, either in the red or the white cells. The leucocytes in the course of the disease are variable in number, but in all cases a slight leucocytosis occurs with the onset of the terminal stage, varying from 10,000 to 27,000. Leucocytosis also occurs with the paralytic attacks. There is an uniform increase in the percentage of the polymorphonuclear cells throughout, greatest in the terminal stage. The author likens the anæmia to that of other cirrhoses, such as that of the liver, kidney, and of syphilis. He finds difficulty in explaining the disappearance of anæmia in the final stage. It does not appear to be due to concentration of the blood from loss of fluid, since cases of other diseases selected, in which loss of fluid was marked, did not present it as a preagonic phenomenon.

[24] Med. Rec., July 2. [25] Jour. of Hygiene, vol. iv., p. 73, 1904. [26] Amer. Jour. Med. Sc., Dec.

